# MILK CONSUMPTION IN INFANTS UNDER ONE YEAR OF AGE AND VARIABLES ASSOCIATED WITH NON-MATERNAL MILK CONSUMPTION

**DOI:** 10.1590/1984-0462/;2017;35;4;00004

**Published:** 2017

**Authors:** Paula Chuproski Saldan, Sonia Isoyama Venancio, Silvia Regina Dias Medici Saldiva, Daniele Gonçalves Vieira, Débora Falleiros de Mello

**Affiliations:** a Universidade Estadual do Centro-Oeste, Guarapuava, PR, Brasil.; b Instituto de Saúde da Secretaria de Estado da Saúde de São Paulo, São Paulo, SP, Brasil.; c Escola de Enfermagem de Ribeirão Preto, Universidade de São Paulo, Ribeirão Preto, SP, Brasil.

**Keywords:** Human milk substitutes, Infant, Nutrition programs and policies, Substitutos do leite humano, Lactente, Programas e políticas de nutrição e alimentação

## Abstract

**Objective:**

To verify the type of milk consumed by children under one year of age and identify variables associated with non-maternal milk consumption (formula or cow milk).

**Methods:**

Cross-sectional study developed during the 2012 National Vaccination Campaign against Poliomyelitis. The companions of 935 children under one year of age answered a structured questionnaire on the child’s diet in the last 24 hours. The estimates are presented by points, with 95%CI. F-statistics were used to check for differences in the proportion of the types of milk consumption according to the children’s age range (<6 months and 6-11 months) and the association between non-maternal milk consumption and the study variables.

**Results:**

The consumption of maternal milk and child formula was higher for children under six months of age - corresponding to 82.8% (95%CI 78.5-86.3) and 70.4% (95%CI 61.4-78.0), respectively -, whereas the consumption of cow milk was higher among children between 6 and 11 months of age - 74.2% (95%CI 66.5-80.6) -, with differences in the consumption proportions (p<0.0001). The variables associated with higher cow milk consumption were lower maternal education (p<0.0001), the fact that the mother does not have a paid occupation (p=0.0015), child doctor’s appointment in the public health network (p<0.0001) and participation in the Child’s Milk Program (p<0.0001).

**Conclusions:**

The infants received cow’s milk early (before the first year of life), especially children from families with lower socioeconomic levels and children who took part in a specific social program for milk distribuition.

## RESUMO

**Objetivos:**

Verificar o tipo de leite consumido por crianças menores de um ano de idade e identificar variáveis associadas ao consumo de leite não materno (LNM) - fórmula infantil ou leite de vaca (LV).

**Métodos:**

Estudo transversal realizado durante a Campanha Nacional de Vacinação contra Poliomielite 2012. Os acompanhantes de 935 crianças menores de um ano responderam a um questionário estruturado sobre a alimentação da criança nas últimas 24 horas. As estimativas são apresentadas por pontos e intervalo de confiança de 95% (IC95%). Adotou-se a estatística F para verificar diferenças na proporção de consumo dos tipos de leite segundo a faixa etária das crianças (<6 meses e 6-11 meses) e a associação entre o consumo de leite não materno e as variáveis estudadas.

**Resultados:**

O consumo de leite materno e fórmula infantil foi maior entre as crianças menores de 6 meses - sendo 82,8% (IC95% 78,5-86,3) e 70,4% (IC95% 61,4-78,0), respectivamente -, enquanto o de leite de vaca foi maior entre as crianças de 6 a 11 meses - 74,2% (IC95% 66,5-80,6) -, com diferenças nas proporções de consumo (p<0,0001). As variáveis associadas ao maior consumo de leite de vaca foram: menor escolaridade materna (p<0,0001), o fato de a mãe não trabalhar fora (p=0,0015), a criança consultar na rede pública de saúde (p<0,0001) e participar do Programa Leite das Crianças - PLC (p<0,0001).

**Conclusões:**

As crianças receberam leite de vaca precocemente (antes do primeiro ano de vida), em especial aquelas pertencentes às famílias de menor nível socioeconômico e inseridas em programa social específico para recebimento de leite.

## INTRODUCTION

Dietary practices in childhood are extremely important and influence the nutritional status of children, requiring their evaluation and monitoring.^1^ Exclusive maternal breastfeeding (EBF) is recommended until the age of 6 months, and, from that moment on, an additional diet should be initiated, maintaining the breastfeeding until the age of two or mor.^2^ ^,^ ^3^ ^,^ ^4^If EBF is not possible, the indication is that the child receives modified milk (formula). The use of whole cow’s milk (CM) until the age of 12 months is contraindicated due to its allergenic potential, excessive protein content and for being considered a risk factor for iron-deficiency anemia.^5^ ^,^ ^6^ ^,^ ^7^

Despite these recommendations, studies indicate that children aged less than 12 months receive non-maternal milk (NMM), and some of these studies showed the consumption of CM.^8^ ^,^ ^9^ ^,^ ^10^ ^,^ ^11^ ^,^ ^12^ ^,^ ^13^ ^,^ ^14^One literature review pointed out that the determinants of unmodified CM consumption are low maternal schooling and low socioeconomic status.^15^

The Child’s Milk Program (PLC) has been practiced in the state of Paraná since 2003, providing one liter of milk a day to children aged between 6 and 36 months of age, belonging to families with per capita income equal to or lower than half a regional minimum wage. The referred program aims at fighting infant malnutrition and encouraging family agriculture. The milk provided is fluid, pasteurized, with minimum 3% fat content and enriched with iron and vitamins A and D.^16^ ^,^ ^17^

There are few studies about the consumption of types of NMM offered to children aged less than one year, and the variables associated with the consumption of this milk. Therefore, the objectives of this study were to assess the type of milk consumed by children aged less than one year and to identify the variables associated with the intake of NMM (formula or CM).

## METHOD

Cross-sectional study conducted during the 2012 National Vaccination Campaign against Poliomyelitis in Guarapuava, Paraná, Brazil. The study population was a group of children aged less than one year who attended the vaccination posts in the urban and rural areas of the city with their parents or guardians.

With information about the population of children aged less than 1 year, vaccinated in the first stage of the 2011 campaign, the sample size was calculated using the prevalence of EBF in children aged less than 6 months, with an estimation of 40% being aged between 2 and 3 months, according to a local study,^18^ and sampling error of 9%. The sample size estimations were obtained by applying the algebraic expression by Lwanga and Lemeshow^19^ and, afterwards, the 5% non-response adjustment and the design effect (deff)^20^ of 1.4. The final sample size resulted in 1,005 children.

The study used the two-stage cluster sampling.^20^ That is, considering that the children were not distributed evenly in the vaccination posts (clusters), the two-stage selection was adopted, and the probability was proportional to the cluster size. The first stage involved the selection of vaccination posts, and the second stage systematically selected the children waiting in line for the vaccination in each post. Thirty-two vaccination posts were selected, and for each of them we estimated the selection fraction necessary to interview the people in charge of approximately 35 children.

Data collection was conducted between June 11 and 29, 2012, by 118 volunteer students in the courses of Nutrition and Nursing at the local university, who were trained for 4 hours. The data collection instrument was a questionnaire with 67 questions, based on that used in vaccination campaigns from the Breastfeeding and Cities project, from the Health Institute of the Health Secretariat of São Paulo,^21^ adopted by the Ministry of Health to conduct the II Research of Breastfeeding Prevalence in Brazilian Capitals and the Federal District (II PPAM/Capitals and FD), in 2008.^22^ The questions about diet were based on all probable foods - maternal milk (MM), water ,tea, NMM, types of NMM, number of times NMM was consumed, porridge, fruit juice, fruit, salty food (cooked, mush or soup) - that the child consumed the day before the interview (24-hour recall) and were given to the guardians of children before vaccination. The other questions referred to the child, the mother and the health service.

The variables used in this study were: NMM consumption (formula or CM), maternal age (≤19, 20-34 or ≥35 years), primiparity (yes or no), maternal schooling (<8, 8-11 or >11 schooling years), maternal occupation (yes or no), living with the child’s father (yes or no), area of residence (urban or rural), health service the child attends (public network or private/insurance) and participating (yes or nor) of the Children’s milk state program.

The descriptive analysis included the calculation of proportions and 95% confidence intervals (CI) for the types of milk consumed by the children. F-statistics was used to identify the variables associated with the intake of NMM and the differences of proportions between the types of milk consumed, using a 5% significance level. To calculate the probability of consumption of the types of milk (breastfeeding (BF), formula, CM and milk from PLC), the logit analysis was used and estimated, by statistical modelling, the probability of the event in relation to the children’s age in days.^22^ All estimations considered the design effect (survey module). The data analysis was processed in Stata, version 11.1 (Stata Corp., College Station, Texas, USA).

This study was approved by the Research Ethics Committee from the Nursing School in Ribeirão Preto, at Universidade de São Paulo (CEP-EERP/USP), report nº 34.613, from June 11, 2012. This study did not require an informed consent form because it consisted of a fast questionnaire applied in the waiting line of the vaccination post. Therefore, the parents or guardians were asked for a verbal consent, in order to not disrupt the vaccination campaign. This procedure was established in the research of the Breastfeeding and Cities project from the Health Institute of the Secretariat of Health in São Paulo and in the II PPAM/Capitals and FD.^21^ ^,^ ^22^

## RESULTS

The participants eligible for the study accounted for 1,118, however, 18 children were excluded for not living in Guarapuava. Sixteen questionnaires were excluded due to inconsistencies in the child’s age, and there were 149 refusals.

Of the 935 children who participated in the study, 459 (49.1%) were aged less than 6 months, and 476 (50.9%) were aged 6 months or more; 470 (50.3%) were female, 476 (50.9%) were born from vaginal delivery, and 853 (91.2%) presented with weight at birth ≥ 2,500 grams. Of the total number of children, 86.7% were accompanied by their mothers at the time of the interview. Of the mothers accompanying their children, 45.8% were primiparous; 57.8% were aged between 20 and 24 years; 50.4% had between 8 and 11 schooling years; 61.5% did not work outside the household; and 65.3% took their children to be cared for in the public health network, as described in Table 1.

**Table 1:** Sociodemographic characteristics of mothers and children aged less than one year participating in the study in Guarapuava (PR), Brazil, 2012.

	n	%
Maternal age (years)^a^		
≤19	195	20.8
20-34	540	57.8
≥35	93	10.0
Not informed^b^	107	11.4
First child^a^		
Yes	428	45.8
No	403	43.1
Not informed^b^	104	11.1
Maternal schooling (years)^a^		
<8	234	25.0
8-11	471	50.4
>11	126	13.5
Not informed^b^	104	11.1
Maternal occupation^a^		
Works outside the household	255	27.3
Does not work outside the household	575	61.5
Not informed^b^	105	11.2
Living with the child’s father^a^		
Yes	715	76.5
No	116	12.4
Not informed^b^	104	11.1
Area of residence		
Urban	839	89.7
Rural	96	10.3
Health service		
Public	611	65.3
Private/insurance	301	32.2
Not informed^b^	23	2.5

^a^Data collected when the children were with their mothers. ^b^Data not answered by the children’s tutor.

Table 2 shows the proportions of milk consumption by the children. There were higher levels of consumption of BF and formula by children aged less than 6 months of age, and CM and PLC milk by children aged from 6 to 11 months, with different proportions in consumption for all types of milk analyzed. The frequency of daily consumption of NMM (formula and/or CM) was one for 13.7% (95%CI 10.6-17.6) of the children, two for 15.6% (95%CI 12.4-19.3) and three or more for 70.7% (95%CI 65.8-75.1).

**Table 2:** Proportion of the consumption of milk by children aged less than one year in Guarapuava (PR), Brazil, 2012.

Types of milk	% of consumption and 95%CI		p-value
	Children <6 months	Children aged 6 to 12 months	
Maternal milk	82.8 (78.5-86.3)	54.5 (48.7-60.2)	<0.0001
Non maternal milk^a^	37.3 (31.8-43.3)	73.0 (68.0-77.5)	<0.0001
Maternal milk + non maternal milk^a^	55.0 (44.9-64.7)	39.9 (34.1-46.0)	0.0133
Formula	70.4 (61.4-78.0)	25.8 (19.4-33.4)	<0.0001
Cow’s milk^b^	29.6 (22.0-38.6)	74.2 (66.5-80.6)	<0.0001
Children’s Milk Program^c^	1.2 (0.3-5.1)	33.7 (25.8-42.7)	<0.0001

^a^Formula and cow’s milk. ^b^Pasteurized milk, long-life type, milk from the Children’s Milk Program and powdered milk. ^c^Milk provided by the government of the State of Paraná to families with per capita income lower than half a regional minimum wage. 95%CI: 95% confidence interval.

**Figure 1:**
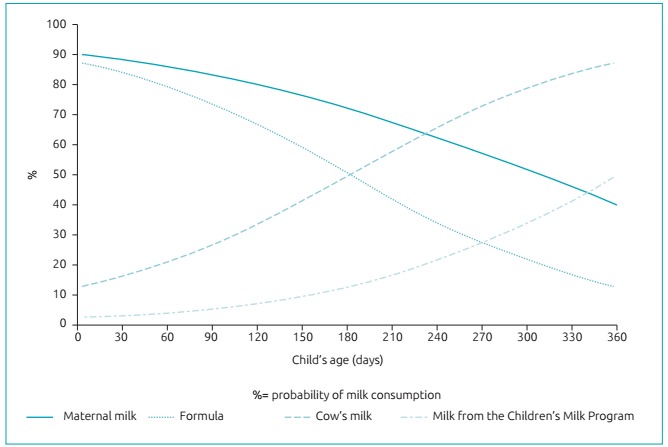
Probability of milk consumption by children aged less than one year in Guarapuava (PR), Brazil, 2012.

Figure 1 shows that BF is high in the first days of life (90%), decreasing to 70% at the age of 6 months and falling to 40% at the end of the first year of life. The formula is also consumed by many children in the first days of life (87%), however, its consumption reduces more expressively than BF. CM is consumed by 16% of the children in the first month of life, increasing to 50% at the age of 6 months, and reaching approximately 90% of the consumption in the end of the first year of life. The consumption of PLC milk increases after the age of 6 months (13%), reaching 50% at the age of 12 months.

The variables associated with higher consumption of CM were: lower maternal schooling - with tendency to increase the consumption with the reduction of maternal schooling -, the fact that the mother did not work outside the household, the child being assisted in the public health network and participating in PLC (Table 3).

**Table 3:** Proportion of consumption of milk according to the variables analyzed in Guarapuava (PR), Brazil, 2012.

	% consumption		p-value
	Formula	Cow’s milk^**a**^	
Maternal age (years)			
≤19	33.0	67.0	0.1643
20-34	43.1	56.9	
≥35	46.0	54.0	
First child			
Yes	45.7	54.3	0.1143
No	36.1	63.9	
Maternal Schooling (years)			
<8	29.0	71.0	<0.0001^b^
8-11	38.1	61.9	
>11	71.0	29.0	
Maternal Occupation			
Yes	50.6	49.4	0.0015
No	35.3	64.7	
Living with the child’s father			
Yes	41.8	58.2	0.6499
No	38.1	61.9	
Area of residence			
Urban	40.7	59.3	0.8604
Rural	39.6	60.4	
Health service			
Public	30.9	69.1	<0.0001
Private/Insurance	57.3	42.7	
Participating in PLC			
Yes	9.9	90.1	<0.0001
Noo	52.3	47.7

^a^Pasteurized, long life milk, in natura, milk from the Children’s Milk Program and powdered milk. ^b^Statistically signiciant liner tendency test. PLC: Children’s Milk Program.

## DISCUSSION

Regarding the research design, it is important to mention that the conduction of surveys in vaccination campaigns has been used as a strategy to obtain data of populations in a short period of time, at low cost.^21^ ^,^ ^23^ The external validity of the study can be assessed by the high coverage of the 2012 Vaccination Campaign against Poliomyelitis in the city, which reached 100% of the children aged less than one year, and by the similar profile of the sample analyzed with the data from the Live Birth Information System (SINASC) in 2011 for the city.^24^ ^,^ ^25^ Of the children studied, 50.9% were born from vaginal delivery, and 91.2% weighed ≥ 2,500 grams *versus* 50.5% and 90.9% of the population of reference (SINASC 2011), respectively.^25^ Regarding the maternal data, 63.9% of the mothers had 8 or more schooling years versus 60.2% of the population of reference (SINASC 2011).^25^

This study showed that BF (82.8%) and the consumption of formula (70.4%) was higher among children aged less than 6 months, whereas CM (74.2%) and PLC milk (33.7%) were more consumed by children aged between 6 and 11 months. The frequency of the daily consumption of NMM (formula and/or CM) was three times or more for 70.7% of the children analyzed. The intake of CM was high among the studied children, which is opposite to the recommendation of national and international institutions, which do not indicate the intake of this type of milk before the 12 months of life due to its allergenic potential, the protein overload and the high chances of the child developing iron deficiency anemia.^6^ ^,^ ^7^ ^,^ ^26^

It is important to mention that the PLC milk offered to children aged from 6 to 36 months, belonging to families with per capita income lower than half a minimum wage, is the whole CM enriched with iron and vitamins A and D.^17^ Therefore, the government of the State of Paraná is going against the recommendations for the consumption of CM, so this state program could be revised and discussed with academic nutrition and pediatrics institutions regarding the provision of CM, which is recommended only after the child is 1 year old, given the implications to the children’s health. Such aspects suggest that this milk may be influencing the non-continuity of BF, since only 39% of the children breast fed at the end of their first year of life.

The suggestion is to conduct a review in the age group of the infant audience to be impacted by PLC, from 6 to 36 months of age to 12 to 36 months of age, besides state and municipal actions defending BF, in order to continue this practice in the first years of life of the children. The relevance of PLC for the rural development and for small and medium-sized producers of the state is recognized and is not being discussed in this paper. However, it is important to think about the implications of CM consumption before the 12 months of life. Another aspect that should be further discussed in this program concerns one of the objectives that elucidates the fight against infant malnutrition, but nowadays the reality points to increasing excess weight among children.

A cross-sectional study that analyzed the consumption of milk among Brazilian children aged less than 60 months found that 91.8% of the ones aged less than 6 months were breastfeeding, and 23% used formula; 74.6% of the children aged from 6 to 12 months received CM, and, in the South region, in this age group, 70% consumed more than two milky meals.^8^ These findings are similar as to the intake of CM and the frequency of milky meals, however, they are different regarding the formula. In a prospective study that assessed the dietary practices of children aged from 4 to 12 months in three Brazilian capitals, the intake of CM among children aged from 6 to 11 months was 77.7%,^11^ similar to that reported in this study. The use of formula among those aged less than 6 months was 12%,^11^ which is below our finding. A cross-sectional study conducted in Acrelândia (AC), Brazil, with children aged from 6 to 24 months, showed that the intake of CM was 70.4% and 77.4% for children aged 6 to 8 months and 9 to 11 months, respectively,^10^ and these values are similar to the ones in this study.

The probability of children being breastfed and receiving formula in the first days of life was high (around 90%), however, the intake of these types of milk decreases until the end of the first year of life, being more expressive for formula (12%). The chances of CM consumption is inverse, so 50% of the children consumed this milk at the age of 6 months. The consumption of PLC milk increased after the age of 6 months, since after this period the children enrolled in the program start receiving the benefit.^17^ This aspect suggests that the consumption of this type of milk before that age can be related to the fact that other children in the family received the benefit, so the mother uses the milk for all children, regardless of age.

A cross-sectional study that analyzed the dietary practices of children aged from 6 to 12 months from 136 cities in the State of São Paulo found that, at the age of 6 months, the probability of a child receiving other types of milk was 70%, and, at the age of 12 months, 83%. However, there is no difference between the types of milk consumed.^27^ Another cross-sectional study that analyzed the regional influence on the early consumption of foods other than BF among children aged less than 6 months in Brazilian capitals and the Federal District emphasized the consumption of tea, mostly in the South region, and milk, in the Southeast and Northeast regions.^9^ This regional behavior can be distinguished after the age of six months, when children from Paraná state are able to apply for PLC, showing the increasing consumption of this type of milk.

This study showed that the socioeconomic level influenced the type of milk consumed by the children, since the variables associated with the higher consumption of CM were lower maternal schooling, the fact that the mother did not work outside the household and participating in PLC. There was also a tendency for the increasing consumption of CM with the reduced maternal schooling. The children who attended public health services also consumed more CM. The findings in this study corroborate those of a review article that points low maternal schooling and low socioeconomic status as determinants for the use of unmodified CM.^15^ Even though the children’s family income was not investigated in this study, maternal schooling was used as a proxy for the socioeconomic status of the families.^9^ A cohort study with children aged less than six months accompanied in the first, fourth and sixth months of life in Viçosa (MG), Brazil, showed that the low income was a risk factor for the consumption of CM in the sixth month of life.^28^

Another study observed that mother with less than eight schooling years, who worked outside the household, had more chances of offering foods from the milk group, including for children aged less than six months.^12^ An investigation showed that the consumption of milky meals (NMM and porridge) was associated with the fact that the mother worked outside the household and had more than eight schooling years.^27^ Another study verified that the introduction of NMM was delayed for mothers who did not work outside the household.^29^ The main findings in these investigations are opposite to those in this study, however, it is worth to mention that the type of NMM consumed was not investigated in the two last studies analyzed. In our study, the fact that the mother did not work outside the household could mean lower income, suggesting that the family with lower per capita income participates in the PLC.

Even though the literature does not show the role of the health service interfering in the type of milk consumed by the children, in this study, the children participating in the PLC should be accompanied every month (weight verification) in the health united as a condition of the program. This suggests an explanation for the fact that the higher intake of CM took place among children who attended the public health network. However, it is worth to mention that despite the association found between the intake of CM and the public health service, this result should be interpreted carefully. A cross-sectional study about the prevalence of breastfeeding in children aged less than two years in Campinas (SP), Brazil, showed that the public health service can have a positive impact on early weaning when compared to the private health system.^30^ A study that accompanied children aged less than six months in the first, fourth, and sixth months of life found that the lower number of prenatal appointments was a risk factor for the intake of CM in all months assessed. The authors conclude that this finding can be owed to the lower access of mothers to information about adequate dietary practices, usually approached in groups addressed to pregnant women in health units.^28^

The importance of BF is consensus in the literature, and children in families with lower socioeconomic status can benefit more from BF, given the impossibility of acquiring formula due to its high cost. The Ministry of Health recommends that if the possibilities of relactation to maintain BF are finished, and at the financial impossibility to acquire formula, professionals should advise mothers as to the adequate and safe use of CM.^3^

Some limitations of this study are the non-investigation of how the types of milk consumed were prepared, that is, if they were offered pure, diluted or with the addition of other foods; and the fact that the data were obtained in a single 24-hour recall. The strong aspect of this study highlights the sampling technique used. Further studies are required to verify the impact of the milk offered in the PLC on the nutritional status of the children who receive this benefit, and the influence of the consumption of this milk in the continuity of BF.

In this study, the children received CM before the first year of life, especially those from families with lower socioeconomic status inserted in the social program to receive milk.
